# A public health response to the methamphetamine epidemic: the implementation of contingency management to treat methamphetamine dependence

**DOI:** 10.1186/1471-2458-6-214

**Published:** 2006-08-18

**Authors:** Steven Shoptaw, Jeffrey D Klausner, Cathy J Reback, Stephen Tierney, John Stansell, C Bradley Hare, Steven Gibson, Michael Siever, William D King, Uyen Kao, Jeffrey Dang

**Affiliations:** 1 David Geffen School of Medicine at UCLA, Department of Family Medicine, Los Angeles, CA, USA; 2 David Geffen School of Medicine at UCLA, Department of Psychiatry, Integrated Substance Abuse Programs, Los Angeles, CA, USA; 3 San Francisco Public Health Department, San Francisco, CA, USA; 4 Friends Research Institute, Inc, Los Angeles, CA, USA; 5 Van Ness Recovery House, Prevention Division, Los Angeles, CA, USA; 6 San Francisco AIDS Foundation, San Francisco, CA, USA; 7 University of California, San Francisco, Positive Health Program, CA, USA; 8 University of California, San Francisco, MAGNET, San Francisco, CA, USA; 9 University of California, San Francisco, Department of Psychiatry, CA, USA

## Abstract

**Background:**

In response to increases in methamphatemine-associated sexually transmitted diseases, the San Francisco Department of Public Health implemented a contingency management (CM) field program called the Positive Reinforcement Opportunity Project (PROP).

**Methods:**

Methamphetamine-using men who have sex with men (MSM) in San Francisco qualified for PROP following expressed interest in the program, provision of an observed urine sample that tested positive for methamphetamine metabolites and self-report of recent methamphetamine use. For 12 weeks, PROP participants provided observed urine samples on Mondays, Wednesdays and Fridays and received vouchers of increasing value for each consecutive sample that tested negative to metabolites of methamphetamine. Vouchers were exchanged for goods and services that promoted a healthy lifestyle. No cash was provided. Primary outcomes included acceptability (number of enrollments/time), impact (clinical response to treatment and cost-effectiveness as cost per patient treated).

**Results:**

Enrollment in PROP was brisk indicating its acceptability. During the first 10 months of operation, 143 men sought treatment and of these 77.6% were HIV-infected. Of those screened, 111 began CM treatment and averaged 15 (42%) methamphetamine-free urine samples out of a possible 36 samples during the 12-week treatment period; 60% completed 4 weeks of treatment; 48% 8 weeks and 30% 12 weeks. Across all participants, an average of $159 (SD = $165) in vouchers or 35.1% of the maximum possible ($453) was provided for these participants. The average cost per participant of the 143 treated was $800.

**Conclusion:**

Clinical responses to CM in PROP were similar to CM delivered in drug treatment programs, supporting the adaptability and effectiveness of CM to non-traditional drug treatment settings. Costs were reasonable and less than or comparable to other methamphetamine outpatient treatment programs. Further expansion of programs like PROP could address the increasing need for acceptable, feasible and cost-effective methamphetamine treatment in this group with exceptionally high rates of HIV-infection.

## Background

Methamphetamine abuse is increasingly one of the most serious public health problems in the United States. Among men who have sex with men (MSM), including both gay and bisexual men, methamphetamine is a popular street drug [1,2], especially along the West Coast. Prevalence rates for methamphetamine use in the previous 6 months among MSM in San Francisco range between 11%–17% [3]. This level of drug use causes concern among public health professionals because of the obvious problems of escalating abuse and dependence. Even more troubling is the fact that methamphetamine use is strongly associated with increased sexual risk behaviors and sexually transmitted diseases (STDs) and HIV-transmission in MSM [4-6]. Interventions that reduce or eliminate methamphetamine use for MSM in drug treatment settings show corresponding and concomitant reductions in sexual risk behaviors that increase STD/HIV transmission and, as such, may represent an important component of a comprehensive STD/HIV control strategy for MSM [7].

A promising intervention for producing at least short-term reductions in methamphetamine use is contingency management. Contingency management (CM) is an efficacious behavioral therapy that provides immediate positive reinforcement – cash incentives or cash equivalents – in exchange for biological evidence of drug abstinence [8,9]. The treatment program can be implemented by staff with minimal clinical training and by itself can produce significant short-term reductions in methamphetamine use in treatment-seeking methamphetamine-dependent men [7]. This article describes the implementation and brief evaluation of a CM treatment program in response to increases in methamphetamine-associated STDs including HIV infections by the San Francisco Department of Public Health.

## Methods

### Participants

In the fall of 2004 the San Francisco Public Health Department fielded a program of contingency management for methamphetamine-using MSM in order to decrease methamphetamine-associated sexual risk behaviors leading to the spread of STDs and HIV infection. The Positive Reinforcement Opportunity Project (PROP, [10]) was started in three health services settings: San Francisco General Hospital out-patient AIDS Ward, Continuum HIV Day Services, and MAGNET, a full-service STD/HIV prevention organization focused on gay men's sexual health. Public Health Department staff in the STD Prevention and Control Section managed the program. That staff included a program coordinator and a health worker. Neither had professional experience in substance use treatment.

PROP was promoted by word of mouth and flyers distributed to medical providers serving MSM at risk for methamphetamine abuse. Eligible participants were methamphetamine-using MSM seeking to reduce or eliminate their use of the drug. To enroll in PROP, methamphetamine-using MSM visited one of the sites and staff screened potential participants to determine recent methamphetamine use (any use in the past 7 days or signed referral from a physician documenting current methamphetamine dependence). Those who met the initial eligibility criteria met with a health worker to receive a 15-minute orientation to PROP, to complete brief admission forms describing their drug use, sexual behaviors, and infectious disease history, and to provide a pre-baseline urine sample tested for metabolites of methamphetamine using a radioimmunoassay (MedTox, Phamatech, San Diego, California). Participants were required to have a positive pre-baseline urine test or medical documentation of current methamphetamine dependence to initiate treatment. Methamphetamine-using MSM unable to document current methamphetamine dependence (either with a positive pre-baseline urine test or physician's referral) were not enrolled in PROP.

### Intervention

Enrolled participants met with a health worker on M-W-F between 8–10 am to provide a directly observed urine sample. Participants were awarded vouchers (i.e., credits) that were redeemable for goods or services that promoted a healthy drug-free lifestyle in exchange for urine samples that were free of metabolites of methamphetamine. The reward schedule is shown in Table 1. A rapid reset procedure allowed participants to return to their place in the escalating schedule after producing three consecutive urine samples that were negative for methamphetamine. At no time was cash provided to participants. When participants chose to redeem their vouchers, staff purchased selected items through the Internet or on the telephone using a credit card. Items were received by mail or made available to the participant for pick-up.

### Measures

Participant characteristics were assessed using a brief baseline survey of recent drug use and sexual risk behaviors. Methamphetamine abstinence was assessed using the urine samples analyzed for the presence of methamphetamine metabolites. Methamphetamine-positive urines and missed appointments were counted as failures. Measures, including the level of cash vouchers accrued, were managed with a computer program specifically created for the project. Costs for implementing PROP were calculated as the total spent on rewards, urine specimen collection and test kits, facility space rental, promotion and the staff time.

While the UCLA Human Subjects Protection Committee provided oversight of the evaluation activities for this public health program, the program itself was non-research and written informed consent was not obtained from participants.

## Results

By the end of the first 10 months of operation (October 2004 to July 2005), 143 unduplicated MSM were eligible and enrolled in the program (14.3 participants per month). As seen in Table 2, participants in the program reported extensive experience with methamphetamine use, with the vast majority reporting at least weekly methamphetamine use. High rates of injection drug use were noted as well as high rates of sexual risk behavior and prior STDs.

Of those 143 who enrolled in PROP, 111 returned for their initial visit and began the CM treatment program. A total of 32 individuals were ineligible for PROP either due to inability to document current methamphetamine dependence or to failure to return to clinic. On average (+/-S.D.) PROP participants produced 15 (+/-13.5) methamphetamine metabolite-free urine samples of the possible 36 samples over the 12-week treatment period. Fifty-seven (52%) achieved 12 metabolite-free urine samples; an additional 19 (17%) were able to provide 24 metabolite-free samples, with only 9 (8.1%) providing all 36 samples free of drug metabolite. Sixty-percent completed 4 weeks of treatment; 48%, 8 weeks and 30%, 12 weeks. The average (+/-S.D.) payout per participant for "reinforcing" methamphetamine free urine samples was $159 (+/-$165) or 35.1% of the possible $453.

The sum of other program costs divided by the number of enrollees was $800.

## Discussion

We successfully implemented a methamphetamine treatment program for methamphetamine-dependent men using CM. Participant characteristics and abstinence outcomes were comparable to MSM who received CM as part of a treatment research program [7]. The 30% retention rate for PROP at the end of 12 weeks is lower than that in studies of CM among methamphetamine-dependent MSM [7] and methamphetamine-dependent heterosexuals [11]. One reason may be that outcomes using CM are sensitive to the richness of the reinforcement schedule [12] and participants in PROP could earn approximately one-third possible in the other CM reports. Despite differences in retention, the average earning per participant in PROP (35.1% of the maximum) is close to that reported in treatment research on the use of CM with methamphetamine-dependent MSM (32.4% of the maximum) [7].

CM was popular among the target community and readily delivered by health department staff with limited training in substance abuse treatment. In San Francisco, methamphetamine-using MSM were willing to comply with the requirement for thrice-weekly observed urine collection in order to reduce their methamphetamine use. Our preliminary findings suggest that the contingency management approach is a feasible and cost-effective means to reduce methamphetamine use in community settings.

The feasibility of PROP is further suggested by the strong participation in the program. One concern is that indigent individuals who do not use methamphetamine may try to "scam" the program in order to access financial benefit. The low rates of complete abstinence in this project do not support that concern. PROP participants report long histories of methamphetamine use with current use indicated by a positive urine drug screen at admission or medical documentation. Further, PROP participants responded to the CM at similar rates in producing metabolite-free urine samples as did treatment-seeking methamphetamine-dependent MSM [7]. If methamphetamine-naïve PROP participants did try to deceptively enter into the program, results indicate they were unable to do it consistently.

The costs of implementing PROP were reasonable. In this community-based program, fixed costs included the contingencies ($17,649), a one-time rent payment ($25,000) and urine test kit costs ($6,700). Staffing required one full-time study coordinator and a part-time assistant totaling $65,000 yearly. The total allocated for this program was $114,349 or a per-capita cost of $800 for the 143 participants who enrolled in PROP. Costs incurred by public health agencies implementing CM for addressing methamphetamine use among MSM could be lessened by contributions from communities and alternative funding agencies.

This report evaluates PROP as a real-world public health program and not a research project, an approach that precluded linking personally identifying information with responses to the intervention, yielding only group-level responses. These are initial outcomes, as only during-treatment data were available, which limits any understanding of the sustained effects of the CM treatment.

## Conclusion

These findings provide valuable information useful in planning community-based interventions for methamphetamine users. This report recounts one of the first adaptations of the CM intervention used outside of specific research or drug-treatment settings. The program was successful in helping methamphetamine-using MSM, a group with an extraordinarily high prevalence of HIV at baseline, to reduce their drug use. The evaluation highlights the flexibility and potency of CM when adapted to address a methamphetamine use problem closely linked to HIV transmission via sexual and drug-related behaviors (injection use) in an urban community of MSM. To the extent that CM reduces episodes of methamphetamine use and concomitant risky sexual behaviors, significant improvements may accrue as indicated by more organized lives for participants and by fewer potential transmission risk events within a community experiencing conjoined methamphetamine and HIV epidemics. Costs for implementing this CM program are modest and within ranges worth considering when selecting interventions to address drug-associated STD and HIV transmission in the context of a comprehensive prevention strategy. PROP continues to be implemented by the San Francisco Public Health Department and wider availability of CM may provide for the necessary expansion in treatment required to address the burgeoning methamphetamine epidemic in groups of users at high risk for HIV transmission.

There are limitations to this report. The most important of these is that no comparison condition was included, which precludes statements of causality. It is possible that some or all of the outcomes were due to factors that arise in delivering PROP. As well, PROP implemented CM as a clinical program, and as such, the evaluation lacks the rigor and control (e.g., experimental design, psychometrically sound measures) common to clinical trials of behavioral therapies. Still the research literature is replete with information on the efficacy of CM for reducing stimulant use. The challenge to the fields of research and public health practice now involves finding ways to adapt and to use this efficacious behavioral intervention to reduce drug use and associated HIV-related transmission behaviors in communities facing these interwoven public health problems.

## Competing interests

The author(s) declare that they have no competing interests.

## Authors' contributions

S. Shoptaw and J. Klausner conceived of this project. J. Klausner was instrumental in finding resources for the project and S. Shoptaw advised its implementation; both were instrumental in writing this evaluation. C.J. Reback and S. Tierney were also instrumental in the conception of the project and in writing the evaluation report. J Stansell, C. Hare, M. Siever and S. Gibson were crucial in the running of the program and in the final editing of this report. W. King was instrumental in writing the initial drafts and in editing the final report, as was U. Kao. J. Dang conducted the statistical analyses and contributed to the writing of the final report. There are 11 authors to this paper due to the collaborative nature of the program of evaluation. Hence all authors had crucial input to this field action report and meet the threshold for authorship in this collaborative effort.

## Pre-publication history

The pre-publication history for this paper can be accessed here:



## Figures and Tables

**Table 1:** Schedule of incentives for the contingency management program used in PROP

	**Monday**	**Wednesday**	**Friday**	**Bonus**	**Weekly totals**
**Week 1**	$2.50	$3.75	$5.00	$10.00	$21.25
**Week 2**	$6.25	$7.50	$8.75	$10.00	$32.50
**Week 3**	$10.00	$10.00	$10.00	$10.00	$40.00
**Week 4**	$10.00	$10.00	$10.00	$10.00	$40.00
**Week 5**	$10.00	$10.00	$10.00	$10.00	$40.00
**Week 6**	$10.00	$10.00	$10.00	$10.00	$40.00
**Week 7**	$10.00	$10.00	$10.00	$10.00	$40.00
**Week 8**	$10.00	$10.00	$10.00	$10.00	$40.00
**Week 9**	$10.00	$10.00	$10.00	$10.00	$40.00
**Week 10**	$10.00	$10.00	$10.00	$10.00	$40.00
**Week 11**	$10.00	$10.00	$10.00	$10.00	$40.00
**Week 12**	$10.00	$10.00	$10.00	$10.00	$40.00
**Grand total**					$453.75

**Table 2:** Drug use, sexual behavior, and sexually transmitted disease history in methamphetamine-using men who have sex with men at entry in the Positive Reinforcement Opportunity Project, San Francisco, 2004–2005.

**N = 143**	**Percent(n)**
**Drug use responses**	
Methamphetamine use	
Daily	42.7% (61)
Weekly	43.4% (62)
Monthly or less	13.9% (20)
Years prior methamphetamine use	
0 to 2 years	17.5% (25)
3 to 5 years	25.9% (37)
6 to 10 years	18.9% (27)
More than 10 years	37.8% (54)
Route of administration^1^	
Smoke	69.9% (100)
Intranasal	67.1% (96)
Inject	54.5% (78)
Rectal	40.6% (58)
**Sexual behavior responses**	
Use of methamphetamine with sex	
Yes	88.1% (126)
No	11.2% (16)
Sexual partners, past month	
None	18.2% (26)
1–2	40.6% (58)
3–5	18.9% (27)
6 or more	22.4% (32)
Sexual behaviors, past month^1^	
Unprotected insertive anal sex	70.6% (101)
Unprotected receptive anal sex	67.8% (97)
Unprotected insertive vaginal sex	16.1% (23)
Sexually transmitted disease history	
Syphilis, past year	20.3% (29)
Gonorrhea, past month	32.9% (47)
Chlamydia, past month	15.4% (22)
HIV status	
Positive	77.6% (111)
Negative	21.7% (31)

^1 ^Participants could endorse more than one response to this item.
